# Bioactive Aliphatic Sulfates from Marine Invertebrates

**DOI:** 10.3390/md17090527

**Published:** 2019-09-09

**Authors:** Luis C. Kellner Filho, Bruno W. Picão, Marcio L. A. Silva, Wilson R. Cunha, Patricia M. Pauletti, Gustavo M. Dias, Brent R. Copp, Camila S. Bertanha, Ana H. Januario

**Affiliations:** 1 Center for Research in Exact and Technological Sciences, University of Franca, Franca, São Paulo 14404-600, Brazil; 2 Center for Natural and Human Sciences, Federal University of ABC, São Bernardo do Campo, São Paulo 09606-070, Brazil; 3 School of Chemical Sciences, University of Auckland, Auckland 1142, New Zealand

**Keywords:** alkyl sulfate, aliphatic sulfate, alkane sulfate, alkene sulfate, marine invertebrates, bioactivity, NMR data

## Abstract

The occurrence of sulfated steroids and phenolics in marine organisms is quite widespread, being typically reported from Echinoderms. In contrast, alkane and alkene aliphatic sulfates are considerably rarer with examples being reported from a diverse array of organisms including echinoderms, sponges and ascidians. While no ecological roles for these metabolites have been proposed, they do exhibit a diverse array of biological activities including thrombin inhibition; the ability to induce metamorphosis in larvae; antiproliferative, antibacterial and antifungal properties; and metalloproteinase inhibition. Of particular interest and an avenue for future development is the finding of antifouling properties with low or nontoxic effects to the environment. This review focuses on alkyl sulfates and related sulfamates, their structures and biological activities. Spectroscopic and spectrometric techniques that can be used to recognize the presence of sulfate groups are also discussed, data for which will enhance the ability of researchers to recognize this class of chemically- and biologically-interesting marine natural products.

## 1. Introduction

Sulfur is the sixth most abundant element and its metabolism is critically important to global biogeochemical cycles. Sulfate is a universal electron acceptor in marine environments because of its high abundance and stability in seawater [1]. Sulfated compounds belonging to the classes of triterpenes, steroids and phenolics are widely distributed in marine organisms with sulfated steroids constituting the largest subset of these secondary metabolites [2,3]. The echinoderms including ophiuroids, sea stars, sea cucumbers and sea urchins are a rich source of sulfated metabolites, especially sulfated saponins (steroidal and triterpenoid) and sulfated aglycone steroids. Ophiuroids are characterized by containing polar sulfated steroidal polyols and a lack of saponins [4,5] while, in marine sponges, these metabolites are frequently found as steroidal and phenolic sulfates [6]. Therefore, the distribution of these compounds is extremely different among the phyla, namely Porifera, Echinodermata, Mollusca and Urochordata, where they have been described [7]. 

However, sulfated alkanes and alkyls are rarer, with only a few aliphatic sulfates having been reported from marine organisms [4,6,8]. These compounds have been isolated from echinoderms, sponges and ascidians, with the examples reported from ascidians being somewhat structurally more complex. The aliphatic sulfated compounds present a wide range of biological activities including morphological-inducing defense [9,10,11,12]; antiviral, antibacterial, and antifungal properties [8,13]; cytotoxic action [13,14,15]; antiproliferative properties [16]; thrombin inhibition [17]; metalloproteinase inhibition [9,18]; and antifouling properties [19]. 

In the context of marine natural products, it is unclear why sulfate has been incorporated into their structures. The presence of a sulfate group on a generally long-chain alkyl/alkenyl hydrophobic scaffold would be expected to provide a surfactant characteristic to the natural products. More generally, sulfation is a metabolic strategy to prevent toxicity in different physiological and pathological processes. In this regard, sulfation is considered as a detoxification pathway due to the increased hydrophilicity and enhanced excretion properties of the metabolite [20,21]. Organic sulfur compounds can be further degraded to methanethiol (CH_3_SH) and metabolized to methionine, ultimately releasing H_2_S from sulfur-containing amino acids cysteine and methionine [22]. In a biological context, the addition, and removal, of sulfate is under the control of enzymes. Sulfotransferase enzymes are responsible for addition of a sulfate groups to phenolic and alcohol hydroxyl groups. For removal, an example exists in the marine environment of alkyl sulfates isolated from an ascidian. Fujimoto et al. [23] described alkyl sulfates as a precursor of ascidian flavor in *Halocynthia roretzi*. This ascidian is well known for the peculiar “ascidian” flavor derived from *n*-alcohols with 8, 9 and 10 carbons being the major components. The enzyme alkyl sulfohydrolase is responsible for the alcohols’ liberation in the ascidian; the authors also suggested the alkyl sulfates might be secreted in the digestive juice, as a large amount of free alcohols was found in gut contents [23].

The majority of reports of marine natural products that contain the sulfate group depict the sulfate group as the neutral acid [2]. Given the strongly acidic nature of organosulfates, the occurrence of an electronically neutral acid is unlikely, and in most cases is presumed to exist as a sodium salt [5]. In several cases, alternative counterions, including ammonium, have been reported [5,24].

There is growing evidence from a number of studies that alkyl sulfates can play important ecological roles. Chemical substances released by predators that lead to phenotypic changes in some of their prey are known as kairomones or infochemicals [25,26,27]. The unicellular green alga *Scenedesmus subspicatus* changes its morphology in the presence of a crustacean *Daphnia magna* [9]. This metamorphosis was supposed to be a self-defense mechanism acquired by the green alga and triggered by a kairomone secreted from *D. magna* [9]. Several aliphatic sulfate kairomones were identified from *Daphnia* as a chemical signal playing an important role in the interactions among living organisms in an aquatic environment [10,11,12].

In recent years, researchers’ interest in natural sulfate substances has increased; for example, natural products with a sulfated scaffold have emerged as antifouling agents with low or nontoxic effects to the environment and have inspired new synthetic sulfated compounds [20,28,29]. The sulfated phenolic acid zosteric acid (*p-*sulfated cinnamic acid), isolated from the seagrass *Zostera marina*, can be cited as an antifouling model from the sea [30]. Another interesting example is a combination of the amino acid D-tyrosine and the synthetic “green” biocide tetrakis hydroxymethyl phosphonium sulfate (THPS), which has been found to inhibit the formation of biofilms of the corrosion-causing SRB (sulfate-reducing bacteria) *Desulfovibrio vulgaris* (ATCC 7757) in in vitro tests [31]. 

**Figure 1:**
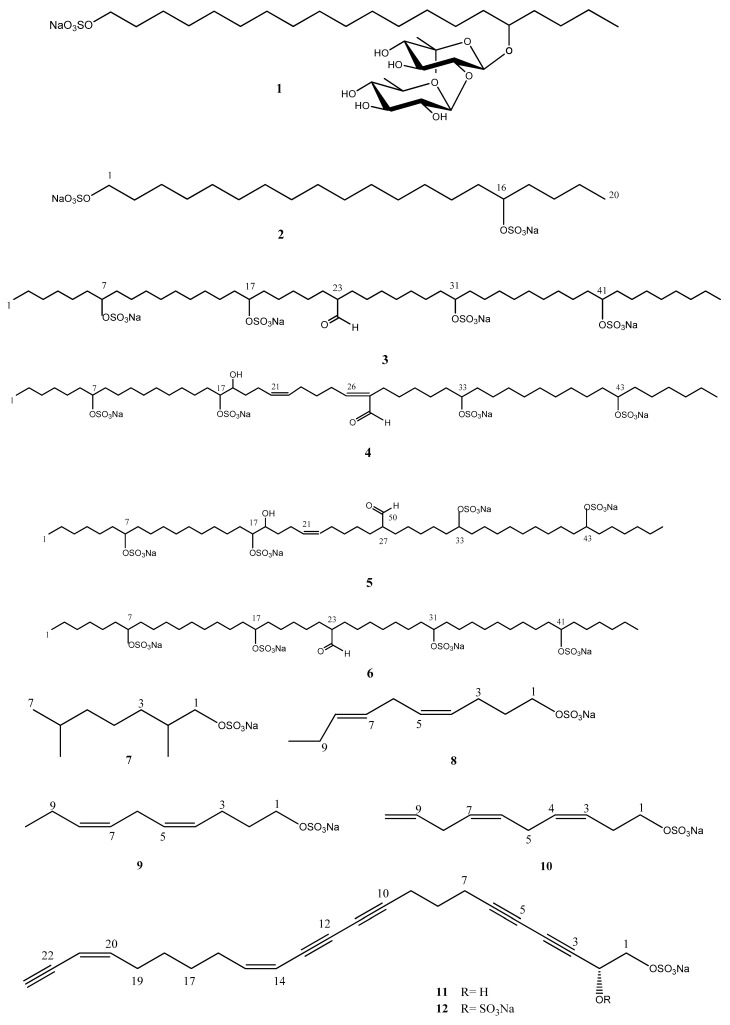

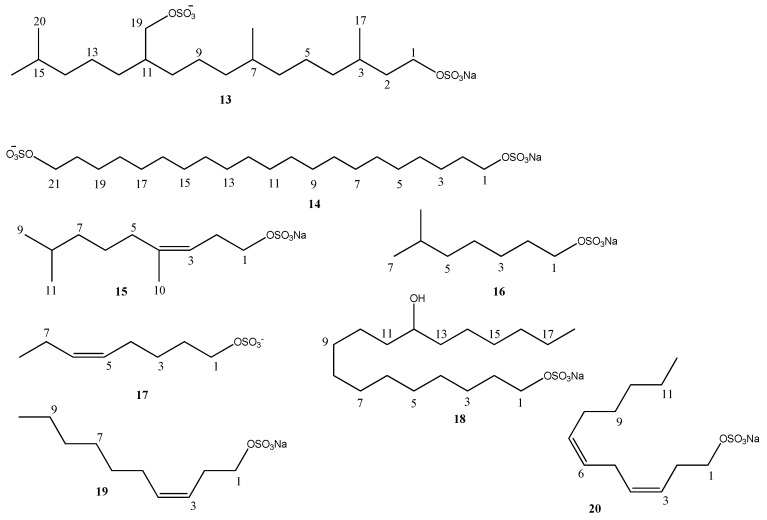
Chemical structure of aliphatic sulfate marine natural products **1–28**.

**Figure 2:**
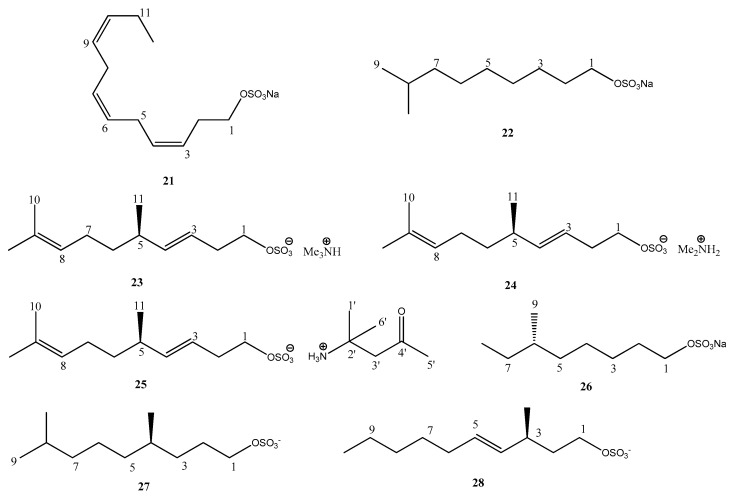

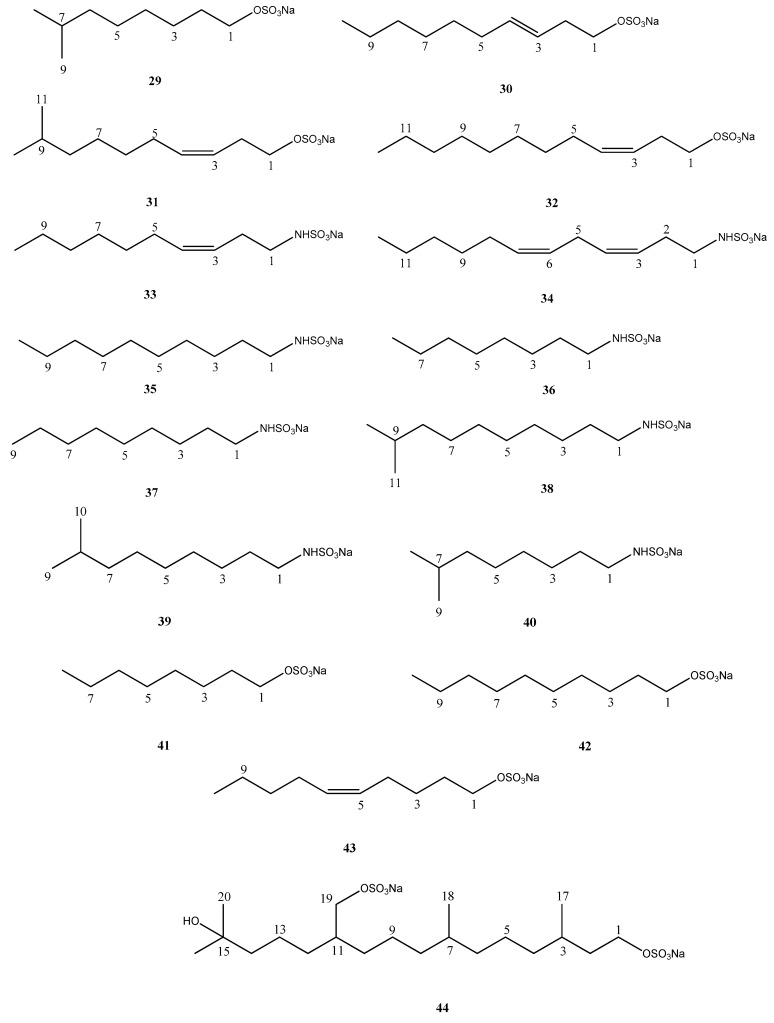

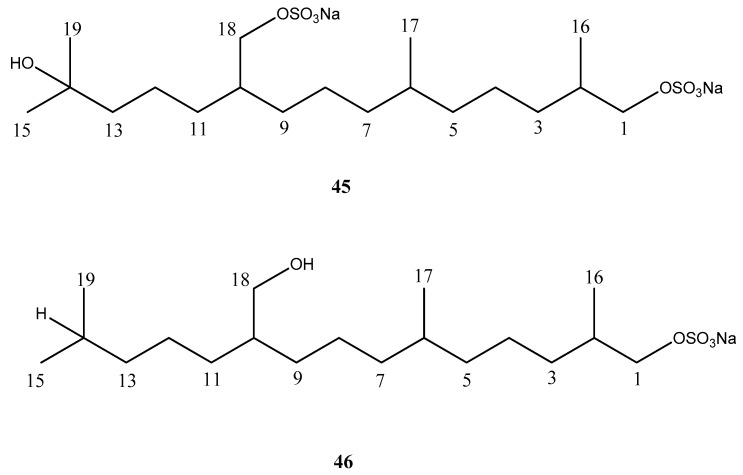
Chemical structure of aliphatic sulfate and sulfamate marine natural products **29–46.**

Nevertheless, the occurrence and distribution of these compounds in marine invertebrates and their biological function remain largely unknown and further studies are required. This review summarizes information on the natural aliphatic sulfates and related sulfamates isolated from marine invertebrates reported to date, of which there are nearly 50, emphasizing their biological potential. The structures of natural products are shown in Figure 1 and Figure 2, and Table 1 lists the reported biological activities. ^1^H and ^13^C-NMR data of the aliphatic sulfates and sulfamates are also summarized. It is anticipated that these data will help facilitate dereplication of these natural product lipids (Table A1, Table A2, Table A3, Table A4, Table A5, Table A6, Table A7, Table A8, Table A9, Table A10, Table A11, Table A12 and Table A13). With the information compiled in this review, our goal is to encourage researchers in the area of marine natural products in search of these rare compounds with a prior knowledge of their biological and ecological potential to extend their research to other biological activities not yet investigated for these metabolites.

## 2. Biological Activity of Aliphatic Sulfates Compounds

**Figure 3:**
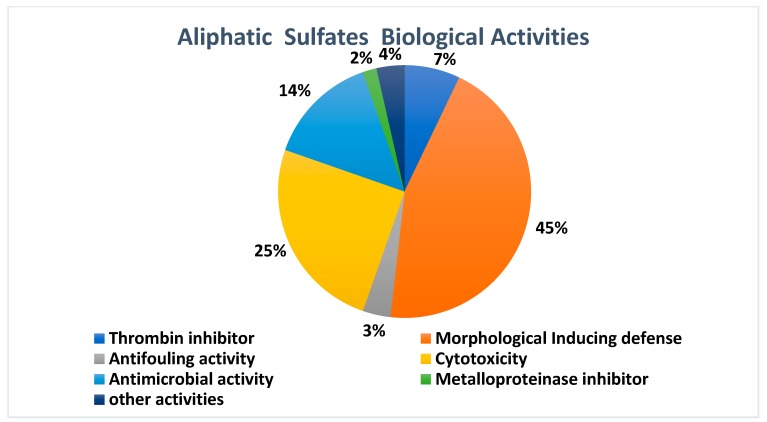
Biological activities of aliphatic sulfates.

Concerning the biological potential of the set of substances analyzed in this review, it was observed that 45% of them showed metamorphosis-inducing activity, highlighting the important role of these substances as kairomones in the interaction with other species. This is followed by cytotoxicity (25%) and antimicrobial (14%) activities (Figure 3). In total, 14 sulfates were tested for in vitro toxicity and the cytotoxicity potential of aliphatic sulfates was expressive against several types of carcinoma. The panel of strains could be expanded and the remaining compounds remain to have an undisclosed cytotoxic potential. The knowledge of the antimicrobial potential of aliphatic sulfates is still insipient, since few strains were investigated and the method used was restricted to zones of growth inhibition measurement. Unfortunately, sulfates **1**, **2**, **23** and **24** have not been tested for any biological activity and, therefore, their biological potential remains unknown. The biological activities of aliphatic sulfates mentioned in this review are outlined in more detail in the following section.

### 2.1. Thrombin Inhibition

The enzyme thrombin is an important target for the treatment of thrombosis and related diseases, which are responsible for deaths and incapacity [36]. The toxadocials A–C and toxadocic acid A (**3**–**6**) were isolated from the marine sponge *Toxadocia cyhdrica.* Toxadocials (**3**–**6**) are a rare class of natural products with one carbaldehyde group in the middle of an alkyl chain. The biosynthesis of these compounds is proposed to involve an aldol condensation of two units of hydroxylated or sulfated aldehydes followed by dehydration and reduction. All four natural products exhibited inhibition of thrombin with IC_50_ of 6.5, 4.6, 3.2 and 2.7 µg/mL, respectively. The authors suggested that the activity was associated with the presence of the sulfate esters. [17,33].

### 2.2. Morphological Inducing Defense

Some sulfated compounds seem to mediate interspecific interactions among aquatic species. According to Yasumoto et al. [9,10,11,12], different sulfated alkanes and alkenes (**7**, **9**, **11**–**12**, **19**–**22**, and **26**–**40**) can induce a morphological defense of phytoplankton. These compounds are known as kairomones since their production by marine grazers works as a semiochemical that induces morphological changes in phytoplankton species including the unicellular *Scenedesmus subspicatus* [37]. When the grazer *Daphnia magna* is present in the water, *S. subspicatus* changes its reproductive mode, forming colonies of two, four and eight cells, which, in addition to the production of spines, are strategies that can increase the chances of *S. subspicatus* escaping consumption.

Life-history changes are also induced in the sea-flea *Daphnia pulex* when exposed to kairomones produced by a predatory midge larvae of the genus *Chaoborus*. Individuals exposed to kairomones produced more defensive structures and released neonates more quickly than control individuals [38]. It is interesting to note that these induced responses were impaired by the presence of waterborne copper 2^+^ ions.

Weiss at al. (2018), in their studies of *Chaoborus* kairomone chemicals inducing defenses in *Daphnia*, found infochemicals from active digestion, consisting of fatty acids conjugated to the amino group of glutamines via the N-terminus. These cues are involved in *Chaoborus* digestive processes, which explains why they are consistently released despite the disadvantage for its emitter [27].

In a somewhat related biological activity, callyspongins A (**11**) and B (**12**), from the sponge *Callyspongia truncate*, showed potent metamorphosis-inducing activity towards the ascidian *Halocynthia roretzi* larvae with ED_100_ values of 0.13 μg/mL and 0.25 μg/mL, respectively [19].

### 2.3. Antifouling

The polyacetylene sulfated callyspongins A (**11**) and B (**12**) showed potent antifouling activity against the barnacle *Balanus amphitrite*, with ED_50_ values of 3.9 and 4.1 µg/mL [19]. According to Quian and co-authors, AF compounds have medium to high bioactivity with a threshold of EC_50_ < 15 µg/mL, and AF compounds that have high LC_50_/EC_50_ ratios (>15) are potentially good candidate antifoulants [30].

### 2.4. Cytotoxicity

Marine natural products can exhibit high levels of cytotoxic activities. In general, alkyl sulfates isolated from marine ascidians have simple structures derived from polyketides and, in some cases, derived from isoprene, with frequently associated cytotoxic and antiproliferative activities [15]. The normoterpenoid **7**, and the sulfates **16** and **17** have been described as cytotoxic towards the murine fibrosarcoma cell line (WEHI) with IC_50_ 20.9, 15.0 and 12.2 µg/mL, respectively [14]. Compounds **13**, **14** and **15** were evaluated against different types of carcinoma, such as human melanoma (IGR-1), murine macrophage (J774), murine fibrosarcoma (WEHI 164) and murine leukemia (P388) and presented only weak cytotoxicity with IC_50_ between 50 and 370 µg/mL [6,16]. Other sulfate alkenes, such as **25**, were weakly active against leukemia cells (P388) [5]. Furthermore, the sulfated alkanes **45** and **46** were active in BALB/c murine macrophages cells (J774A.1) at 100 µM [15]. The presence of a sulfate group at C-18 and/or the hydroxyl group at the end of the chain of **45** and **46** seems to be essential for the cytotoxic activity, since compounds without these groups did not exhibit biological activity. The chain length also seems to influence activity since compound **45** (C_19_) was more active than compound **44** (C_20_) against cells J774A.1 [15]. In the same way, the sulfates **41**–**43** and **30** isolated from sea cucumber *Apostichopus japonicus* exhibited pronounced cytotoxicity against lung adenocarcinoma human cells line A549 with IC_50_ 0.063, 0.062, 0.064 and 0.065 µM, respectively [13]. The sulfated alkenes (5*Z*)-decenyl sulfate (**43**) and (3*E*)-decenyl sulfate (**30**) were isolated from the sea cucumber *Apostichopus japonicus* and showed potent activity against osteosarcoma cells (MG63) with IC_50_ values of 0.064 and 0.057 µM, respectively [13].

### 2.5. Antimicrobial Activity

Antibacterial and antifungal activities were reported for **7–10**, isolated from the hepatopancreas of the ascidian *Halocynthia roretzi*, with zones of growth inhibition (12 mm and 10 mm) for all the compounds at concentration of 0.2 mg/disk against *Vibrio alginoliticus* and *Mortierella ramaniana*, respectively [8]. The sulfates **41**–**43** and **30** isolated from sea cucumber *Apostichopus japonicus* also showed potent antibacterial action against *Escherichia coli* at 0.05 µg/disk, with compounds **43** and **30** exhibiting zones of growth inhibition of 13 and 15 mm, respectively [13]. Thus, indicating a selective action to Gram-negative bacteria, which has a double phospholipid membrane that protects the cell wall, which rendered the entry to the cell more difficult [39] Additionally, compounds **43** and **30** presented a growth inhibition zone of 8 mm when evaluated against the fungal *Septoria trici*. Those data led to the conclusion that the double bond at the carbon chain improved the biological activity in the case of compounds **43** and **30**, when compared mainly with **42**, which has the same number of carbons.

### 2.6. Metalloproteinase Inhibitor

Metalloproteinases are correlated to various physiological processes, such as extra-cellular matrix degradation and tissue remodeling. Problems with the regulation of these enzymes can lead to pathological conditions, such as cancers, cardiac, cartilage, and neurological problems, making metalloproteinase inhibitors of interest [40]. Sodium 1-(12-hydroxy) octadecanyl sulfate (**18**) was isolated from a marine tunicate of the family Polyclinidae and is structurally related to the forbesins [32]. The natural compound (55:45 mixture of the two isomers 12*R* and 12*S*) and the synthetic **18** inhibited matrix metalloproteinase 2 (MMP2) with an IC_50_ value of 9.0 µg/mL [18].

### 2.7. Other Activities

The alkyl sulfates **7–10** were isolated from the hepatopancreas extract of the ascidian *Halocynthia roretzi*, suggesting that they may play some physiological role in the digestive system of these ascidians. Studies regarding the marine sponge *Callyspongia truncata* led to the isolation of compounds callyspongins A (**11**) and B (**12**). These polyacetylene sulfated compounds inhibited the fertilization of starfish gametes. Compound **11** was particularly potent, inhibiting sperm mobility at 50 µM and prevented fertilization at 6.3 µM. Compound **12** was weaker in its biological activity, inhibiting sperm mobility at 100 µM and blocking fertilization at 50 µM, highlighting the ecological role of these compounds in the interaction with other species [35]. No mode of action studies of **11** and **12** have been reported.

### 2.8. Chemical Characterization of Aliphatic Sulfates

To date, 38 natural alkyl sulfates and eight related sulfamates have been reported from marine invertebrates. In this review, the ^1^H and ^13^C NMR data reported for these natural products are tabulated (Table A1, Table A2, Table A3, Table A4, Table A5, Table A6, Table A7, Table A8, Table A9, Table A10, Table A11, Table A12 and Table A13) to make it easier for research groups to recognize the presence of these metabolites in marine samples. The chemical shifts of hydrogen and carbons near the point of sulfation are characteristically different in sulfated and non-sulfated compounds, allowing the distinction between sulfated compounds and their respective alcohols.

Compounds containing sulfate groups typically have a strong absorption in the IR spectrum at 1350–1450 cm^−1^. In addition, the presence of the sulfate group can be determined by the sodium rhodizonate test. Sodium rhodizonate gives an orange–red colored complex with the Ba^2+^ ion. The absorbance of the red-colored complex is measured at 520 nm. In the presence of sulfate, BaSO_4_ precipitate forms, and, consequently, the color and the absorbance value decrease [41]. Mass spectrometry can also be useful for detection of sulfate, with the presence of the [M − H]^−^ = *m/z* 97 ion detected in negative ion mode ESIMS, suggesting the presence of the sulfate group in the structure.

Another method used to confirm the presence of sulfate substitution is solvolysis to afford the corresponding alcohol [16]. One method for this is to heat the sulfate in dioxane at 100 °C for 4 h [24] or to heat the sulfate in dioxane-piridine mixture (1:1) at 130 °C for 3 h [14]. Subsequent analysis of the alcohol-containing product, particularly concerning adjacent methylene protons, can lead to evidence of the presence of a sulfate group in the original natural product. Methylene groups bearing the sulfate moiety exhibited an increased ^1^H NMR chemical shift when compared to the chemical shifts of the corresponding alcohol. The combination of this downfield shift of methylene ^1^H NMR signals and a higher than expected polarity of the original sample may be indicative of the presence of a primary alcohol esterified by sulfuric acid [4].

For example, the ^1^H-NMR and ^13^C-NMR spectra of compound **15** showed a methylene group bearing the sulfate group at δ 3.99 (2H, H-1), and δc 69.0 in contrast with the respective alcohol signal at δ 3.65 (2H, H-1) [6]. In a similar manner, the ^1^H-NMR spectrum of 6-methylheptyl sulfate (**16**) presents a triplet associated to the methylene group bearing the sulfate moiety at δ 4.02 (2H, H1, *J*= 6.8), but, after solvolysis of **16**, the corresponding alcohol displayed the upfield shifted signal at δ 3.63 [14].

Compound **1**, named forbesin, was isolated from *Asterias forbesi* Desor and has a differentiated chemical skeleton due to the glycolipidic aliphatic sulfated chain. The sugar unit is composed of two 6-*β*-linked quinovose units; however, no biological activity was associated with it. The alkane **2** was also obtained from the same source [32]. Among the 46 chemical structures shown in this review, 24 compounds have a saturated carbon chain (**1**–**7, 13, 14, 16, 18, 22, 26**–**29, 41**–**42,** and **44**–**46**) and six of them belong to the sulfamate class, correlated to sulfated hydrocarbons (**35**–**40**).

Considering the unsaturated compounds, 10 of them have a single double bond (**5, 15, 17, 19, 28, 30**–**32,** and **43**), as well as the sulfamate **33**. Eight structures pointed to having two double bonds in their skeletons, these being the sulfates **4**, **8**–**9, 20,** and **23**–**25**, including the sulfamate **34**. Compounds **11** and **12** are distinguished by the presence of five triple bonds and are polyacetylene sulfates.

On the other hand, the chemical structures of compounds **2**–**6, 11**–**14,** and **44**–**45** called attention to the presence of more than one sulfate group in the main carbon chain.

By comparing the lengths of the carbon chains, compounds **16** and **41** are found to have the smallest chain—containing eight carbons atoms—contrasting with the longer carbon chains with 50 C-atoms present in compounds **2**–**4**.

2,6 dimethylheptylsulfate (**7**) was the first nor-monoterpenoid isolated from the tunicate *Polycitor Adriaticus* collected in the Northern Adriatic Sea, [α]_D_ + 4.7° (*c* = 0.01 MeOH) [37]. This compound was also isolated from the hepatopancreas from the ascidian *Halocynthia roretzi* as a racemate [α]_D_ 0° (*c* = 0.40 MeOH) [10]. In 2006, Yasumoto and co-authors isolated compound **7** from *Daphinia*, and the absolute configuration was determined by the ^1^H-NMR analysis of the (*R*)-α-methoxy-α-(trifluoromethyl) phenylacetic acid (MTPA) ester of **7**. The authors additionally synthesized the 2(*R*) and 2(*S*) epimers of **7** to compare their induction of a morphological defense of phytoplankton, but no difference was found between the two sulfates [10].

Compounds **23**–**25**, isolated from the Echinus *Temnopleurus hardiwickii*, present the peculiar presence of both sulfate and ammonium ions; while sulfated alkenes are common in marine invertebrates, the occurrence of complex counterions are rare [5]. The trimethylammonium moiety in **23** was confirmed by HRESIMS. The *E* configuration of the double bond in C-3 was assigned on the basis of the H-3/H-4 coupling constant (15.4 Hz) and the ^13^C NMR chemical shifts of C-2 (δ 32.6) and C-5 (δ 36.2). The absolute configuration of 23 was determined through oxidative degradation.

The peculiar polyacetylene sulfates, callyspongins A (**11**) and B (**12**), were isolated from the marine sponge *Callyspongia truncate.* The presence of an ene–diyne unit was suggested by the UV spectrum that showed absorption maxima at 267 and 287 nm (ε = 8900 and 7100, respectively). The absolute configuration of C-2 in **11** was determined to be *R*, after hydrolysis and comparison of the optical rotation data and spectroscopic data of the product obtained with siphonodiol, isolated from *Siphonochalina truncata* [35].

On the other hand, the toxadocials are distinguished by the presence of multiple sulfate units in their chemical structures, as supported by the IR band (1250 cm^−1^), by positive sodium rhodizonate test and by a deshielded methine signal for H-7, H-17, H-31 and H-41 (δ 4.31, quint, *J* = 7 Hz; δ_C_ 81.0) in toxadocial A (**3**), for example. Toxadocial A was also hydrolyzed to afford the respective tetraol.

In addition to the multiple sulfates, the toxadocials have the aldehyde function (δ_H_ 9.51, d, δ_C_ 207.6) together with the alkyl chains [17].

As presented, the chemistry of marine invertebrates, especially the ascidians, has drawn attention due to the peculiarity of its metabolites. Considering sulfated aliphatic molecules, some of these compounds occur in relatively remarkable quantities in ascidians, being a sign of the importance of these metabolites for these organisms and among living organisms in aquatic ecosystems. Consequently, their distribution in a marine environment, quantitative studies involving the biosynthesis of these compounds, the evaluation of the biological potential and the participation of these compounds as kairomones are approaches that need to be better understood, since the contribution of sulfate groups seems to be determinant of the biological activity and interactions between species.

## 3. Conclusions

Alkyl sulfates constitute an interesting group of marine natural products that have been reported from organisms from a diverse range of Phyla (Echinodermata, Porifera, Chordata and Mollusca). The natural products cover a structural diversity that encompasses differences in stereochemistry, chain length, the presence of unsaturation and number of sulfates present in the molecular structure. The association of different biological activities with this structural class make them of particular interest. Among these properties, the ability of alkyl sulfates to exhibit ecologically-relevant morphological, metamorphosis-inducing and antifouling activities makes them attractive for further study. The relative ease of detection of the presence of the sulfate substituent(s), via negative ion ESI MS and IR spectroscopy, combined with microscale hydrolysis to the corresponding alcohol and subsequent 1H NMR chemical shift comparative analysis, means these rare marine natural products should be more readily recognized in chromatography column fractions by natural product chemists. In this respect, the compilation of NMR data may be valuable for a quick confirmation of the presence of these compounds in marine species. The discovery of new examples of alkyl sulfate natural products, combined with synthesis, will accelerate the ability to undertake further studies and perhaps uncover new biological functions for this specific class of metabolites.

## Figures and Tables

**Table 1:** Summary of biological activities reported for aliphatic sulfate and sulfamate marine natural products.

Bioactivity	Compound	Species	Reference
No activity	Forbesin (**1**)	*Asterias forbesi*	[32]
No activity	Dissulfato 5 (**2**)	*Asterias forbesi*	[32]
**Trombin inhibitor**			
	Toxadocial A (**3**)	*Toxadocia cylindrica*	[17]
	Toxadocial B (**4**)	*Toxadocia cylindrica*	[33]
	Toxadocial C (**5**)	*Toxadocia cylindrica*	[33]
	Toxadocic acid A (**6**)	*Toxadocia cylindrica*	[33]
**Morphological-inducing defense**			
	2,6-dimethylheptyl sulfate (**7**)	*Daphnia*	[10,14]
	(4 *Z*,7 *Z*)-4,7-decadienyl sulfate (**9**)	*Daphnia pulex*	[12]
	callyspongin A (**11**)	*Callyspongia truncata*	[19]
	callyspongin B (**12**)	*Callyspongia truncata*	[19]
	3-Decen-1-ol, 1-(hydrogen sulfate), (3*Z*) (**19**)	*Daphinia pulex*	[9]
	3,6-Dodecadien-1-ol, 1-(hydrogen sulfate), (3*Z*,6*Z*)- (**20**)	*Daphinia pulex*	
	3,6,9-Dodecatrien-1-ol, 1-(hydrogen sulfate), (3*Z*,6*Z*,9*Z*)- (**21**)	*Daphinia pulex*	
	1-Nonanol, 8-methyl-, 1- (hydrogen sulfate) (**22**)	*Daphinia pulex*	
No activity	trimethylammonium (5R)-5,9-dimethyl-(3*E*)-3,8-decadienyl-1-sulfate (**23**)	*Temnopleurus hardwickii*	[5]
No activity	dimethylammonium (5R)-5,9-dimethyl-(3E)-3,8-decadienyl-1-sulfate (**24**)	*Temnopleurus hardwickii*	
	(S)-6-methyloctyl sulfate (**26**)	*Daphnia*	[10]
	4(*R*),8-Dimethylnonyl Sulfate (**27**)	*Daphnia*	[11]
	3(*S*)-Methyl-4*E*-decenyl Sulfate (**28**)	*Daphnia*	
	7-methyloctyl sulfate (**29**)	*Daphnia pulex*	[12]
	3*E*-decenyl sulfate (**30**)	*Daphnia pulex*	
	9-methyl-3*Z*-decenyl sulfate (**31**)	*Daphnia pulex*	
	3*Z*-dodecenyl sulfate (**32**)	*Daphnia pulex*	
	3*Z*-decenyl sulfamate (**33**)	*Daphnia pulex*	
	3,6-dodecadienyl sulfamate (**34**)	*Daphnia pulex*	
	decyl sulfamate (**35**)	*Daphnia pulex*	
	octyl sulfamate (**36**)	*Daphnia pulex*	
	nonyl sulfamate (**37**)	*Daphnia pulex*	
	9-methyldecyl sulfamate (**38**)	*Daphnia pulex*	
	8-methylnonyl sulfamate (**39**)	*Daphnia pulex*	
	7-methyloctyl sulfamate (**40**)	*Daphnia pulex*	
**Antifouling Activity**			
	callyspongin A (**11**)	*Callyspongia truncata*	[19]
	callyspongin B (**12**)	*Callyspongia truncata*	[19]
**Cytotoxicity**			
	2,6-dimethylheptyl sulfate (**7**)		[14]
	3,7-dimethyl-15-isopropyl-11- -hexadecyl sulfate (**13**)	*Ascidia mentula*	[16]
	monosodium mono(henicosane-1,21-diyl bis(sulfate)) (**14**)	*Ascidia mentula*	[16]
	(3*Z*)-4,8-dimethylnon-3-en-l-sulfate (**15**)	*Microcosmus vulgaris Ophiocoma echinata*	[4,6]
	6-methylheptyl sulfate (**16**)	*Halocynthia papillosa*	[14]
	(*E*)-5-Octenyl sulfate (**17**)	*Halocynthia papillosa*	[14]
	2′-methyl-4’-oxobutan-2-ylammonium (5R)-5,9-dimethyl-(3E)-3,8-decadienyl-1-sulfate (**25**)	*Temnopleurus hardwickii*	[5]
	octyl sulfate (**41**)	*Apostichopus japonicus*	[13]
	decyl sulfate (**42**)	*Apostichopus japonicus*	
	(5*Z*)-dec-5-en-1-yl sulfate (**43**)	*Apostichopus japonicus*	
	3*E*-decenyl sulfate (**30**)	*Apostichopus japonicus*	
	1,15-Hexadecanediol, 3,7,15-trimethyl-11-[(sulfooxy)methyl]-, 1-(hydrogen sulfate), sodium salt (1:2) (**44**)	*Aplidium elegans*	[15]
	-1,14-Pentadecanediol, 2,6,14-trimethyl-10-[(sulfooxy)methyl]-, 1-(hydrogen sulfate), sodium salt (1:2) (**45**)	*Aplidium elegans*	
	1,11-Undecanediol, 2,6-dimethyl-10-(4-methylpentyl)-, 1-(hydrogen sulfate), sodium salt (1:1) (**46**)	*Aplidium edwardisii*	
**Antimicrobial**			
	2,6-dimethylheptyl sulfate (**7**)	*Halocynthia roretzi Policitor adriaticus*	[8,34]
	(4*Z*,7*E*)-4,7-decadienyl sulfate (**8**)	*Halocynthia roretzi*	[8]
	(4*Z*,7*Z*)-4,7-decadienyl sulfate (**9**)	*Halocynthia roretzi*	[8]
	(3*Z*,6*Z*)-3,6,9-decatrienyl sulfate (**10**)	*Halocynthia roretzi*	[8]
	octyl sulfate (**41**)	*Apostichopus japonicus*	[13]
	decyl sulfate (**42**)	*Apostichopus japonicus*	[13]
	(5*Z*)- decenyl sulfate (**43**)	*Apostichopus japonicus*	[13]
	3*E*-decenyl sulfate (**30**)	*Apostichopus japonicus*	[13]
**Metalloproteinase 2 (MMP2) inhibitor**			
	sodium 1-(12-hydroxy) octadecanyl sulfate (**18**)	Polyclinidade	[18]
**Inhibition of starfish fertilization**			
	callyspongin A (**11**)	*Callyspongia truncata*	[35]
	callyspongin B (**12**)	*Callyspongia truncata*	[35]

## Appendix A

**Table A1:** ^13^C chemical shifts (δ in ppm) of sulfates **1**, **3** and **4**. Ref. = References.

Compounds	1 ^1^		3	4		3	4
Solvent	E	Solvent	E	E	Solvent	E	E
Ref.	[32]	Ref.	[17]	[33]	Ref.	[17]	[33]
Carbon	δ_C_	Carbon	δ_C_	δ_C_	Carbon	δ_C_	δ_C_
1	67.3	1	14.4	14.20	26	30.8	155.33
16	78.8	2	23.7	22.84	27	30.8	143.97
20	12.7	3	-	32.04	28	30.8	24.12
1′	102.8	4	30.5	29.81	29	26.0	28.99
2′	81.4	5	26.0	25.47	30	35.3	~30
3′	74.3 ^a^	6	35.3	35.02	31	81	25.20
4′	74.1 ^a^	7	81	78.78	32	35.3	34.82
5′	70.5 ^b^	8	35.3	34.93	33	26.0	78.88
6′	16.4	9	26.0	25.47	34	30.8	34.93
1′’	99.6	10	30.8	29.76	35	30.8	25.47
2′’	74.7 ^a^	11	30.8	~30	36	30.8	~30
3′’	75.2 ^a^	12	30.8	~30	37	30.8	~30
4′’	74.1 ^a^	13	30.8	~13	38	30.8	~30
5′’	71.3 ^b^	14	30.8	29.76	39	26.0	~30
6′’	16.5	15	26.0	26.07	40	35.3	29.76
-	-	16	35.3	31.56	41	81.0	25.47
-	-	17	81	82.90	42	35.3	34.93
-	-	18	35.3	72.60	43	26.0	78.78
-	-	19	26.0	32.21	44	30.5	35.02
-	-	20	30.8	24.56	45	33.0	25.47
-	-	21	30.8	131.23	46	23.7	29.81
-	-	22	29.9	129.43	47	14.4	32.04
-	-	23	53.2	27.19	48	207.6	22.84
-	-	24	28.1	28.92	49	-	14.20
-	-	25	30.8	28.70	50	-	195.31

^a,b^ Signals with identical letters may be interchanged. ^1^ The solvent legend is as follows: A = DMSO-*d6;* B = CD_3_OD; C = CDCl_3;_ D = C_5_D_5_N and E = C_5_D_5_N/ H_2_O (5:1)

**Table A2:** ^13^C chemical shifts (δ in ppm) of sulfates **7**–**18**. Ref. = References.

Compounds	7	7	8	9	10	11	12	13	14	15	16	17	18
Solvent	A	B	A	A	A	B	A	B	B	B	B	B	B
Ref.	[8]	[13]	[8]	[8]	[8]	[35]	[35]	[16]	[16]	[6]	[14]	[14]	[18]
Carbon		δ_C_											
1	70.5 (t)	70.1	65.0 (t)	65.3 (t)	65.0 (t)	70.2 (t)	69.1 (t)	67.5	69.1	69.0	68.9	68.9	69.13
2	32.6 (d)	70.1	29.1 (d)	29.2 (d)	27.4 (t)	68.6 (d)	60.5 (d)	37.6	30.5	29.1	30.6	29.6	30.45
3	33.2 (t)	33.8	23.2 (t)	23.3 (t)	126.1 (d)	72.0 (s)	77.1 (s)	30.8	26.8	121.0	26.9	26.5	26.91
4	24.0 (t)	34.1	127.2 (d)	127.2 (d)	126.7 (d)	73.3 (s) ^a^	68.6 (s) ^a^	38.6	30.1	139.4	30.0	27.0	30.72
5	38.8 (t)	25.1	127.9 (d)	128.3 (d)	25.3 (t)	66.3 (s) ^a^	65.2 (s) ^a^	25.3	30.1	32.9	40.3	129.1	30.72
6	274 (d)	39.6	29.8 (t)	25.1 (t)	128.8 (d)	83.2 (s)	80.5 (s)	38.7	30.1	26.8	28.8	132.0	30.72
7	-	-	129.5 (d)	129.2 (d)	129.2 (d)	19.5 (t)	17.7 (t)	34.1	30.1	39.9	23.0	20.9	30.72
8	-	-	131.8 (d)	131.5 (d)	31.0 (t)	28.3 (t)	26.4 (t)	29.0	30.1	29.0	23.0	23.0	30.72
9	-	-	23.2 (t)	20.1 (t)	136.7 (d)	19.8 (t)	18.0 (t)	25.6	30.1	23.0	-	-	30.72
10	-	-	13.7 (q)	14.2 (q)	114.9 (t)	85.3 (s)	84.5 (s)	32.5	30.1	23.6	-	-	26.80
11	-	-	-	-	-	67.3 (s)	65.5 (s)	39.2	30.1	23.0	-	-	38.43
12	-	-	-	-	-	79.4 (s)	78.0 (s)	32.3	30.1	-	-	-	30.1
13	-	-	-	-	-	74.2 (s)	72.5 (s)	25.6	30.1	-	-	-	38.43
14	-	-	-	-	-	109.6 (d)	108.0 (d)	40.5	30.1	-	-	-	26.80
15	-	-	-	-	-	150.0 (d)	148.3 (d)	29.1	30.1	-	-	-	30.72
16	-	-	-	-	-	31.7 (t)	30.0 (t)	23.1	30.1	-	-	-	33.08
17	-	-	-	-	-	29.4 (t)	27.6 (t)	19.7	30.1	-	-	-	23.72
18	-	-	-	-	-	29.4 (t)	27.6 (t)	20.0	30.1	-	-	-	30.1
19	-	-	-	-	-	31.3 (t)	29.5 (t)	71.6	26.8	-	-	-	-
20	-	-	-	-	-	147.6 (d)	145.2 (d)	23.1	30.5	-	-	-	-
21	-	-	-	-	-	109.8 (d)	108.6 (d)	-	69.1	-	-	-	-
22	-	-	-	-	-	82.3 (s)	80.2 (s)	-	-	-	-	-	-
23	-	-	-	-	-	83.8 (d)	84.6 (d)	-	-	-	-	-	-
Me-2	16.9 (q)	17.3	-	-	-	-	-	-	-	-	-	-	-
Me-6 and 7	22.5 e 22.6 (each q)	23.0 and 22.9	-	-	-	-	-	-	-	-	-	-	-

^a^ Signals with identical letters may be interchanged.

**Table A3:** ^13^C chemical shifts (δ in ppm) of sulfates **19**–**32**. Ref. = References.

Compounds	19	20	21	22	23	24	25	27	29	30	31	32		26
Solvent	B	B	B	B	C	C	C	B	B	B	B	B	Solvent	B
Ref.	[9]	[9]	[9]	[9]	[5]	[5]	[5]	[11]	[12]	[12]	[12]	[12]	Ref.	[10]
Carbon	δ_C_												Carbon	δ_C_
1	68.6	68.4	68.4	69.1	67.8	68.3	68.2	69.5	69.2	68.9	68.5	68.6	1	61.9
2	28.5	28.6	28.6	30.5	32.6	32.6	32.5	29.2	30.6	33.7	30.5	28.3	2	30.5
3	125.6	125.9	126.2	26.9	123.1	123.0	123.1	34.2	26.9	126.6	24.5	125.7	3	27.2
4	133.4	131.7	131.4	30.4	139.1	139.4	139.3	33.8	30.5	134.3	132.7 ^a^	133.5	4	27.8
5	28.2	26.6	26.6	31.0	36.2	36.3	36.3	38.4	28.4	33.7	128.4 ^a^	128.4 ^a^	5α	37.7
6	30.7	128.7	128.8	28.5	36.9	37.0	37.0	25.9	40.1	30.5	26.3	30.7 ^a^	5β	37.7
7	30.0	131.3	129.5	40.2	25.6	25.7	25.7	40.5	29.1	29.9	129.8 ^a^	30.6 ^a^	6	35.6
8	32.9	28.1	26.4	29.2	124.5	124.6	124.6	28.1	23.1	32.9	130.1 ^a^	30.4 ^a^	7α	30.6
9	23.7	30.5	128.2	23.1	131.1	131.2	131.2	23.1	23.1	23.7	21.4	30.4 ^a^	7β	30.6
10	14.4	32.6	132.8	23.1	17.6	17.7	17.7	23.0	-	14.4	14.7	33.1	8	11.7
11	-	23.6	21.4	-	20.5	20.5	20.5	-	-	-	-	23.7	9	19.6
12	-	14.4	14.6	-	25.5	25.7	25.7	-	-	-	-	-	-	-
N-CH_3_	-	-	-	-	45.3	35.5	-	-	-	-	-	-	-	-
2′	-	-	-	-	-	-	53.2	-	-	-	-	-	-	-
3′	-	-	-	-	-	-	49.9	-	-	-	-	-	-	-
4′	-	-	-	-	-	-	208.7	-	-	-	-	-	-	-
5′	-	-	-	-	-	-	31.0	-	-	-	-	-	-	-
1′, 6′	-	-	-	-	-	-	25.7	-	-	-	-	-	-	-

^a^ Signals with identical letters may be interchanged.

**Table A4:** ^13^C chemical shifts (δ in ppm) of sulfates **33**–**46** and **30**. Ref. = References.

Compounds	33	35	36	37	40	41	42	43	30		44		45	46
Solvent	B	B	B	B	B	D	D	D	D	Solvent	B	Solvent	B	B
Ref.	[12]	[12]	[12]	[12]	[12]	[13]	[13]	[13]	[13]	Ref.	[15]	Ref.	[15]	[15]
Carbon	δ_C_									Carbon	δ_C_	Carbon	δ_C_	
1	44.8	45.0	45.0	45.0	45.0	67.1	67.8	68.1	67.6	1	66.7	-	-	-
2	28.7	30.9 ^a^	30.9 ^a^	30.9 ^a^	30.8	32.1	32.2	29.8	33.5	2α	36.7	1 β	73.2	73.6
3	127.5	28.2	28.3	28.2	28.3	30.2	30.2	26.6	126.5	2β	36.7	2	33.5	34.1
4	132.9	30.7 ^a^	30.5 ^a^	30.7 ^a^	30.8	29.7	30.1	27.3	133.0	3	29.9	3	33.7	34.3
5	28.3	30.7 ^a^	30.4 ^a^	30.6 ^a^	28.5	29.6	30.0	130.1	33.0	4	37.6	4	24.5	25.1
6	30.7	30.6 ^a^	33.0	30.4 ^a^	40.2	26.5	29.8	130.5	29.7	5	24.6	5	37.6	38.1
7	30.0	30.5 ^a^	23.7	33.1	29.2	23.0	29.7	27.4	29.2	6α	37.6	6	33.0	33.5
8	32.9	33.1	14.5	23.7	23.0	14.4	26.5	32.4	32.0	6β	37.6	7	37.6	38.1
9	23.7	23.7	-	14.5	23.0	-	23.1	22.8	23.0	7	33.1	8	24.5	25.1
10	14.4	14.5	-	-	-	-	14.4	14.4	14.4	8α	37.7	9	31.7	31.9
11	-	-	-	-	-	-	-	-	-	8β	37.7	10	38.4	41.4
12	-	-	-	-	-	-	-	-	-	9	24.6	11	31.9	31.9
-	-	-	-	-	-	-	-	-	-	10	32.0	12	24.5	25.2
-	-	-	-	-	-	-	-	-	-	11	38.4	13	44.4	40.3
-	-	-	-	-	-	-	-	-	-	12	31.7	14	70.7	28.9
-	-	-	-	-	-	-	-	-	-	13	24.6	15	28.3	22.8
-	-	-	-	-	-	-	-	-	-	14	44.4	16	16.3	16.9
-	-	-	-	-	-	-	-	-	-	15	70.8	17	19.2	19.9
-	-	-	-	-	-	-	-	-	-	16	28.4	18	70.8	65.5
-	-	-	-	-	-	-	-	-	-	17	18.9	19	28.3	22.8

^a^ Signals with identical letters may be interchanged.

**Table A5:** ^1^H chemical shifts (δ in ppm) of sulfates **1**–**4**. Ref. = References.

Compounds	1		3	4		3	4
Solvent	E		A	A		A	A
Ref.	[32]		[17]	[33]		[17]	[33]
Hydrogen	δ_H_, m, *J* (Hz)	Hydrogen	δ_H_, m, *J* (Hz)	δ_H_, m, *J* (Hz)	Hydrogen	δ_H_, m, *J* (Hz)	δ_H_, m, *J* (Hz)
1	4.53 (*J* = 6.7)	1	0.89 (t, *J* = 6.7)	0.77 (t, *J* = 6.8)	26	1.3 (m)	6.43 (t, *J* = 7.4)
16	3.75	2	1.3 (m)	1.15 (m)	27	1.3 (m)	-
20	0.95 (*J* = 7.0)	3	1.3 (m)	1.15 (m)	28	1.3 (m)	2.29 (t, *J* = 7.4)
1′	5.18 (*J* = 7.6)	4	1.3 (m)	1.16 (m)	29	1.63–1.46(m), ~1.39 (m)	1.39 (quint, *J* = 7.4)
2′	4.05 ^a^	5	1.63–1.46(m) ~1.39 (m)	1.53 (m)	30	1.65 (m)	1.25 (m)
3′	4.26 (*J* = 8.6)	6	1.65 (m)	1.91 (m), 1.81(m)	31	4.31 (quint, *J* = 4.9)	1.53 (m)
4′	3.77 ^b^	7	4.31 (quint, *J* = 4.9)	4.88 (quint, *J* = 5.9)	32	1.65 (m)	1.91 (m) 1.81 (m)
5′	3.73 ^b^	8	1.65 (m)	1.91 (m) 1.81 (m)	33	1.63–1.46(m) ~1.39 (m)	4.86(quint, *J* = 5.9)
6′	1.63 (*J* = 6.2)	9	1.63–1.46(m) ~1.39 (m)	1.53 (m)	34	1.3 (m)	1.91 (m) 1.81 (m)
1′’	4.81 (*J* = 7.6)	10	1.3 (m)	1.25 (m)	35	1.3 (m)	1.53 (m)
2′’	4.09 ^a^	11	1.3 (m)	1.16 (m)	36	1.3 (m)	1.25(m)
3′’	4.17 (*J* = 9.7)	12	1.3 (m)	1.16 (m)	37	1.3 (m)	1.16 (m)
4′’	3.89^b^	13	1.3 (m)	1.16 (m)	38	1.3 (m)	1.16 (m)
5′’	3.80 ^b^	14	1.3 (m)	1.25 (m)	39	1.63–1.46(m) ~1.39 (m)	1.16 (m)
6′’	1.65 (*J* = 6.2)	15	1.63–1.46(m) ~1.39 (m)	1.73 (m) 1.55 (m)	40	1.65 (m)	1.25 (m)
-	-	16	1.65 (m)	2.02 (m) 1.71 (m)	41	4.31 (quint, *J* = 4.9)	1.53 (m)
-	-	17	4.31 (quint, *J* = 4.9)	5.08	42	1.65 (m)	1.91 (m) 1.81 (m)
-	-	18	1.65 (m)	4.28 (dt, *J* = 10.0, 2.5)	43	1.63–1.46(m) ~1.39 (m)	4.88 (quint, *J* = 5.9)
-	-	19	1.63–1.46 (m) ~1.39 (m)	2.01 (m) 1.77 (m)	44	1.3 (m)	1.91 (m) 1.81 (m)
-	-	20	1.3 (m)	2.55 (dq, *J*=14.3, 7.3) 2.39 (m)	45	1.3 (m)	1.53 (m)
21	-	21	-	5.57 (dt, *J*= 10.5, 7.3)	46	1.3 (m)	1.16 (m)
22	-	22		5.44 (dt, *J*= 10.5,7.3)	47	0.89 (t, *J* = 6.7)	1.15 (m)
23	-	23	2.22 (m)	2.16 (q, 7.3)	48	9.51(d, *J* = 3.0)	1.15 (m)
Me-2	-	24	1.3 (m)	1.50 (quint, *J*=7.3)	49	-	0.77 (t, *J* = 6.8)
Me-6 and 7	-	25	1.3 (m)	2.30 (q, *J*=7.4)	50	-	9.50 (s)

^a.^ Values with the same superscript may be interchanged.

**Table A6:** ^1^H chemical shifts (δ in ppm) of sulfates **7**–**10**. Ref. = Reference.

Compounds	7		7	8	9	10	
Solvent	A		B	A	A	A	
Ref.	[8]		[13]	[8]	[8]	[8]	
Hydrogen		δ_H_, m, *J* (Hz)					
1	3.45 (dd, *J* = 9.5, 6.8) 3.54 (dd, *J* = 9.5, 5.9)		4.29 (dd, *J* = 8.0, 8.2) 4.40 (dd, *J* = 9.1, 6.1)	3.67 (m)	3.71 (m)	3.68 (m)	
2	1.61 (m)		1.90–1.96 (m)	1.53 (m)	1.54 (m)	2.27 (m)	
3	1.00 (m) 1.28 (m)		1.29–1.35 (m) 1.05–1.11 (m)	2.02 (m)	2.05 (m)	5.35 (m)	
4	1.20 (m) 1.27 (m)		1.29–1.35 (m) 1.25–1.28 (m)	5.36 (m)	5.27 (m)	5.37 (m)	
5	1.12 (m)		1.11–1.13 (m)	5.36 (m)	5.32 (m)	2.76 (m)	
6	1.50 (septet, *J* = 6.6)		1.48–1.51 (m)	2.69 (m)	2.73 (m)	5.37 (m)	
7	-		-	5.36 (m)	5.32 (m)	5.37 (m)	
8	-		-	5.43 (m)	5.32 (m)	2.79 (m)	
9	-		-	1.96 (m)	2.02 (m)	5.78 (dd, *J* = 17.0, 10.0)	
10	-		-	0.91 (t, *J* = 7.0)	0.91 (t, *J* = 7.0)	4.96 (dd, *J* = 10.0, 1.8) 5.03 (dd, *J* = 17.0, 1.8)	
Me-2	0.82 (d, *J* = 6.9)		0.87 (d, *J* = 6.6)	-	-	-	
Me-6 and 7	0.84 (d, *J* = 6.6)		1.03 (d, *J* = 6.6) 0.87 (d, *J* = 6.6)	-	-	-	
2-OH	-		-	-	-	-	

**Table A7:** ^1^H chemical shifts (δ in ppm) of sulfates **11**–**17**. Ref. = References.

Compounds	11	12	13	14	15	16	17
Solvent	B	A	B	B	B	B	B
Ref.	[35]	[35]	[16]	[16]	[6]	[14]	[14]
Hydrogen	δ_H_, m, *J* (Hz)						
1	4.14 (d, *J* = 5.5	3.68 (m)	4.07 (m)	4.02 (t, *J* = 6.0)	3.99 (t, *J* = 7.0)	4.02 (t, *J* = 6.8)	4.01 (t, *J* = 6.8)
2	5.20 (t, *J* = 5.5)	4.43 (m)	1.48^b^ (Ha)	4.02 (t, *J* = 6.0)	2.40 (bq, *J* = 7.0)	1.69 (m)	1.65 (m)
3	-	-	1.65 ^b^	1.69 (m)	5.19 (bt)	1.43 (m)	1.45 (m)
4	-	-	1.35 ^b^	1.42 (m)	-	1.32 (m)	2.15 (m)
5	-	-	1.33-1.40 ^b^	1.32 (large signal)	2.06 (t, *J* = 7.5)	1.23 (m)	5.23 (dt, *J* = 11, 0, 6.8)
6	-	-	1.32 ^b^	1.32 (large signal)	1.43 (m)	1.58 (m)	5.40 (dt, *J* = 11, 0, 6.8)
7	2.44 (t, *J* = 6.9)	2.38 (t, *J* = 6.9)	1.45 ^b^	1.32 (large signal)	1.21 (m)	0.92 (t, *J* = 6.5)	2.08 (m)
8	1.75 (quint, *J* = 6.9)	1.67 (quint, *J* = 6.9)	1.14 (m, Ha) 1.35 ^b^ (Hb)	1.32 (large signal)	1.57 (m)	0.92 (t, *J* = 6.5)	0.99 (t, *J* = 7.0)
9	2.45 (t, *J* = 6.9)	2.42 (t, *J* = 6.9)	1.33–1.40 ^b^	1.32 (large signal)	0.91 (d, *J* = 6.5)	-	-
10	-	-	1.31 ^b^	1.32 (large signal)	1.72 (bs, *J* = 7.0)	-	-
11	-	-	1.67 ^b^	1.32 (large signal)	0.91 (d, *J* = 6.5)	-	-
12	-	-	1.35 ^b^	1.32 (large signal)	-	-	-
13	-	-	1.33-1.40 ^b^	1.32 (large signal)	-	-	-
14	5.52 (d, *J* = 11.0)	5.56 (d, *J* = 11.0)	1.22 (m)	1.32 (large signal)	-	-	-
15	6.14 (dt, *J* = 11.0, 7.3)	6.12 (dt, *J* = 11.0, 7.8)	1.58 (m)	1.32 (large signal)	-	-	-
16	2.31 (m)	2.22 (m)	0.91 (d, *J* = 6.5)	1.32 (large signal)	-	-	-
17	1.44 (m)	1.35 (m)	0.96 (d, *J* = 6.5)	1.32 (large signal)	-	-	-
18	1.44 (m)	1.35 (m)	0.90 (d, *J* = 6.5)	1.32 (large signal)	-	-	-
19	2.32 (m)	2.23 (m)	3.94 (d, *J* = 5.0)	1.32 (large signal)	-	-	-
20	6.04 (dt, *J* = 11.0, 7.8)	5.99 (dt, *J* = 11.0, 7.8)	0.91 (d, *J* = 6.5)	1.42 (m)	-	-	-
21	5.48 brd (*J* = 11.0)	5.46 (dd, *J* = 11.0, 1.5)	-	1.69 (m)	-	-	-
22	-	-	-	-	-	-	-
23	3.46 (brs)	3.95 (d, *J* = 1.5)	-	-	-	-	-
2-OH	-	5.68 (brs)	-	-	-	-	-

^a^ Values with the same superscript may be interchanged. ^b^ Signals overlapped by other resonances.

**Table A8:** ^1^H chemical shifts (δ in ppm) of sulfates **18**–**22**. Ref. = References.

Compounds	18	Compounds	19	20	21	22
Solvent	B	Solvent	B	B	B	B
Ref.	[18]	Ref.	[9]	[9]	[9]	[9]
Hydrogen	δ_H_, m, *J* (Hz)	Hydrogen	δ_H_, m, *J* (Hz)			
1	3.99 (t, *J* = 6.5)	1	4.00 (t, *J* = 7.0)	4.02 (t, *J* = 7.0)	4.03 (t, *J* = 7.0)	4.03 (t, *J* = 7.0)
2	1.54 (dt, *J* = 15.0, 6.7)	2	2.46 (q, *J* = 7.0)	2.49 (q, *J* = 7.0)	2.50 (q, *J* = 7.0)	1.70 (q, *J* = 7.0)
3	1.38 (m)	3	5.44 (dt, *J* = 11.0, 7.0)	5.45 (dt, *J* = 11.0, 7.0)	5.45–5.54 (m)	1.50–1.40 (m)
4 ^b^	1.28–1.34	4	5.53 (dt, *J* = 11.0, 7.0)	5.47 (dt, *J* = 11.0, 7.0)	5.45–5.54 (m)	1.47–1.30 (envelop)
5 ^b^	1.28–1.34	5	2.11 (q, *J* = 7.0)	2.86 (t, *J* = 7.0)	2.90 (t, *J*=5.0)	1.47–1.30 (envelop)
6 ^b^	1.28–1.34	6	1.38 (overlapped)	5.36 (dt, *J* = 11.0 and 7.0)	5.30–5.45 (m)	1.47–1.30 (envelop)
7 ^b^	1.28–1.34	7	1.34 (envelop)	5.42 (dt, *J* = 11.0 and 7.0)	5.30–5.45 (m)	1.22 (m)
8 ^b^	1.28–1.34	8	1.34 (envelop)	2.12 (q, *J* = 7.0)	2.86 (t, *J* = 5.0)	1.57 (nonet, *J* = 7.0)
9 ^b^	1.28–1.34	9	1.34 (envelop)	1.40 (overlapped)	5.30–5.45 (m)	0.92 (d, *J* = 7.0)
10	1.31 ^c^	10	0.94 (t, *J*=7.0)	1.37 (envelop)	5.30–5.45 (m)	0.92 (d, *J* = 7.0)
11	1.42 (m)	11	-	1.37 (envelop)	1.37 (envelop)	-
12	3.49 (quint, *J* = 3.75)	12	-	0.96 (t, *J* = 7.0)	1.01 (t, *J* = 7.0)	-
13	1.42 (m)	N-CH_3_	-	-	-	-
14	1.31	2′	-	-	-	-
15 ^b^	1.28–1.34	3′	-	-	-	-
16	1.29 ^c^	4′	-	-	-	-
17	1.32 ^c^	5′	-	-	-	-
18	0.90 (t, *J* = 7.1)	1′, 6′	-	-	-	-

^b^ Methylene envelope. ^c^ Overlapping on methylene envelope.

**Table A9:** ^1^H chemical shifts (δ in ppm) of sulfates **23**–**26**. Ref. = References.

Compounds	23	24	25		26
Solvent	C	C	C	Solvent	B
Ref.	[5]	[5]	[5]	Ref.	[10]
Hydrogen	δ_H_, m, *J* (Hz)			Hydrogen	δ_H_, m, *J* (Hz)
1	4.02 (t, *J* = 7.3)	4.02 (t, *J* = 7.1)	4.01 (t, *J* = 7.1)	1	4.03 (t, *J* = 6.6)
2	2.37 (m)	2.39 (m)	2.38 (m)	2	1.70 (quint, *J* = 6.6)
3	5.36 (dt, *J* = 15.4, 6.1)	5.34 (dt, *J* = 15.4, 6.4)	5.34 (dt, *J* = 15.4, 6.5)	3	1.48–1.29 (m)
4	5.38 (dd, *J* = 15.4, 7.0)	5.40 (dd, *J* = 15.4, 7.3)	5.39 (dd, *J* = 15.4, 7.0)	4	1.48–1.29 (m)
5	2.06 (m)	2.07 (m)	2.06 (m)	5α	1.48–1.29 (m)
6	1.27 (m)	1.27 (m)	1.26 (m)	5β	1.24–1.13 (m)
7	1.92 (m)	1.92 (m)	1.92 (m)	6	1.48–1.29 (m)
8	5.07 (t, *J* = 6.5)	5.07 (t, *J* = 6.5)	5.07 (t, *J* = 6.4)	7α	1.48–1.29 (m)
9	-	-	-	7β	1.24–1.13 (m)
10	1.58 (s)	1.58 (s)	1.58 (s)	8	0.92 (t, *J* = 7.8)
11	0.94 (d, *J* = 6.6)	0.95 (d, *J* = 6.9)	0.94 (d, *J* = 6.6)	9	0.91 (d, *J* = 7.4)
12	1.67 (s)	1.68 (s)	1.67 (s)	-	-
NH	9.75 (brs)	8.15 (brs)	7.55 (brs)	-	-
N-CH_3_	2.96 (d, *J*=3.7)	2.77 (t, *J* = 5.5)	-	-	-
2′	-	-	-	-	-
3′	-	-	2.94 (s)	-	-
4′	-	-	-	-	-
5′	-	-	2.21 (s)	-	-
1′, 6′	-	-	1.46 (s)	-	-

**Table A10:** ^1^H chemical shifts (δ in ppm) of sulfates **27**–**31**. Ref. = References.

Compounds	27	28	29	30	31
Solvent	B	B	B	B	B
Ref.	[11]	[11]	[12]	[12]	[12]
Hydrogen					
1	4.02 (t, *J* = 6.6)	4.04 (m)	4.03 (t, *J* = 6.6)	4.01 (t, *J* = 7.1)	4.00 (t, *J* = 7.2)
2	1.70 (m)	1.67 (m)	1.68 (quint, *J* = 6.6)	2.39 (qd, *J* = 6.6, 1.2)	2.46 (q, *J* = 6.8)
3	1.50 (m)	2.31 (sept, *J* = 7.1)	1.48–1.30 (m)	5.47 (dtd, *J* = 15.2, 6.6, 1.2)	5.44 (dt, *J* = 11.5, 7.1)
4	1.50 (m)	5.30 (ddt, *J* = 15.3, 7.9, 1.2)	1.48–1.30 (m)	5.59 (dtd, *J* = 15.2, 6.6, 1.2)	5.52 (dt, *J* = 11.5, 7.1)
5	1.50 (m)	5.48 (dtd, *J* = 15.3, 6.6, 1.0)	1.48–1.30 (m)	2.04 (q, *J* = 6.6)	2.12 (m)
6	1.50 (m)	2.03 (q, *J* = 6.6)	1.23 (m)	1.45–1.30 (envelop)	1.45–1.30 (m)
7	1.50 (m)	1.28–1.44 (m)	1.56 (nonet, *J* = 6.8)	1.45–1.30 (envelop)	1.45–1.30 (m)
8	1.58 (nonet, *J* = 6.6)	1.28–1.44 (m)	0.92 (d, *J* = 6.6)	1.45–1.30 (envelop)	1.25 (m)
9	0.92 (d, *J* = 6.6)	1.28–1.44 (m)	0.92 (d, *J* = 6.6)	1.45–1.30 (envelop)	1.57 (nonet, *J* = 6.6)
10	-	0.93 (t, *J* = 6.8)	-	0.94 (t, *J* = 7.1)	0.92 (d, *J* = 6.6)
11	-	-	-	-	0.92 (d, *J* = 6.6)
Me-8	0.92 (d, *J* = 6.6)	-	-	-	-
Me-3	-	1.04 (d, *J* = 6.8)	-	-	-

**Table A11:** ^1^H chemical shifts (δ in ppm) of sulfates **32**–**39**. Ref. = References.

Compounds	32	33	34	35	36	37	38	39
Solvent	B	B	B	B	B	B	B	B
Ref.	[12]	[12]	[12]	[12]	[12]	[12]	[12]	[12]
Hydrogen	δ_H_, m, *J* (Hz)							
1	4.00 (t, *J* = 7.2)	3.03 (t, *J* = 7.0)	3.04 (t, *J* = 7.0)	3.01 (t, *J* = 7.0)	3.01 (t, *J* = 7.0)	3.01 (t, *J* = 7.0)	2.99 (t, *J* = 7.0)	3.01 (t, *J* = 7.0)
2	2.46 (q, *J* = 6.8)	2.34 (q, *J* = 7.0)	2.37 (q, *J* = 7.0)	1.58 (m)	1.58 (m)	1.58 (m)	1.57 (m)	1.58 (m)
3	5.44 (dt, *J* = 11.0, 7.1)	5.42 (dt, *J* = 11.0, 7.1)	5.47–5.37 (m)	1.34 (envelop)	1.36 (envelop)	1.34 (envelop)	1.33 (envelop)	1.34 (envelop)
4	5.53 (dt, *J* = 11.0, 7.1)	5.50 (dt, *J* = 11.0, 7.0)	5.47–5.37 (m)	1.34 (envelop)	1.36 (envelop)	1.34 (envelop)	1.33 (envelop)	1.34 (envelop)
5	2.10 (m)	2.12 (q, *J* = 7.0)	2.88 (t, *J* = 7.0)	1.34 (envelop)	1.36 (envelop)	1.34 (envelop)	1.33 (envelop)	1.34 (envelop)
6	1.50–1.25 (envelop)	1.37 (overlapped)	5.47–5.37 (m)	1.34 (envelop)	1.36 (envelop)	1.34 (envelop)	1.33 (envelop)	1.34 (envelop)
7	1.50–1.25 (envelop)	1.33 (envelop)	5.47–5.37 (m)	1.34 (envelop)	1.36 (envelop)	1.34 (envelop)	1.33 (envelop)	1.23 (m)
8	1.50–1.25 (envelop)	1.33 (envelop)	2.12 (q, *J* = 7.0)	1.34 (envelop)	0.94 (t, *J* = 7.0)	1.34 (envelop)	1.19 (m)	1.58 (m)
9	1.50–1.25 (envelop)	1.33 (envelop)	1.33 (envelop)	1.34 (envelop)	-	0.94 (t, *J* = 7.0)	1.57 (m)	0.92 (d, *J* = 7.0)
10	1.50–1.25 (envelop)	0.94 (t, *J* = 7.0)	1.33 (envelop)	0.94 (t, *J* = 7.0)	-	-	0.92 (d, *J* = 7.0)	0.92 (d, *J* = 7.0)
11	1.50–1.25 (envelop)	-	1.33 (envelop)	-	-	-	0.92 (d, *J* = 7.0)	-
12	0.94 (t, *J* = 6.6)	-	0.95 (t, *J* = 7.0)	-	-	-	-	-
Me-3	-	-	-	-	-	-	-	-

**Table A12:** ^1^H chemical shifts (δ in ppm) of sulfates **40**–**43** and **30**. Ref. = References.

Compounds	40	41	42	43	30
Solvent	B	D	D	D	D
Ref.	[12]	[13]	[13]	[13]	[13]
Hydrogen	δ_H_, m, *J* (Hz)				
1	3.01 (t, *J* = 7.0)	4.53 (t, *J* = 6.0)	4.55 (t, *J* = 6.0)	4.49 (t, *J* = 6.4)	4.56 (t, *J*= 7.1)
2	1.52–1.63 (m)	1.75–1.82 (m)	1.79–1.85 (m)	1.79–1.84 (m)	2.58–2.59 (m)
3	1.41–1.30 (m)	1.42–1.43 (m)	1.42–1.45 (m)	1.52–1.56 (m)	5.54 (dt, *J* = 15.1, 7.6)
4	1.41–1.30 (m)	1.39–1.41 (m)	1.35–1.23 (overlapped)	2.06–2.08 (m)	5.56 (dt, *J* = 15.1, 7.6)
5	1.41–1.30 (m)	1.35 (m)	1.35–1.23 (overlapped)	5.43 (dt, *J* = 10.5, 6.5)	1.94–1.95 (m)
6	1.24 (m)	1.31–1.32 (m)	1.35–1.23 (overlapped)	5.46 (dt, *J* = 10.5, 6.5)	1.34–1.38 (m)
7	1.52–1.63 (m)	1.20–1.26 (m)	1.35–1.23 (overlapped)	2.06–2.08 (m)	1.30–1.34 (m)
8	0.92 (d, *J* = 7.0)	0.91 (t, *J* = 5.0)	1.35–1.23 (overlapped)	1.38–1.41 (m)	1.27–1.30 (m)
9	0.92 (d, *J* = 7.0)	-	1.35–1.23 (overlapped)	1.36–1.38 (m)	1.25–1.27 (m)
10	-	-	0.94 (t, *J* = 7.0)	0.93 (t, *J* = 7.0)	0.89 (t, *J* = 12.0)
11	-	-	-	-	-
12	-	-	-	-	-

**Table A13:** ^1^H chemical shifts (δ in ppm) of sulfates **44**–**46**. Ref. = References.

Compounds	44	Compounds	45	46
Solvent	B	Solvent	B	B
Ref.	[15]	Ref.	[15]	[15]
Hydrogen	δ_H_, m, *J* (Hz)			
1	4.02 (m)	1 α	3.80 (dd, *J* = 6.6, 9.1)	3.79 (dd, *J* = 6.6, 9.4)
2α	1.42 ^a^	1 β	3.88 (dd, *J* = 6.6, 9.1)	3.87 (dd, *J* = 6.6, 9.4)
2β	1.68 (m)	2	1.78 (m)	1.79 (m)
3	1.60 (m)	3	1.14 (m) 1.40 1.41 ^a^	1.14 (m) 1.43 1.44 ^a^
4	1.10 ^a^ 1.30 1.30 ^a^	4	1.33 ^a^	1.32 ^a^
5	1.30 ^a^	5	1.10 ^a^ 1.30 1.30 ^a^	1.11 ^a^ 1.32 1.30 ^a^
6α	1.10 ^a^	6	1.39 ^a^	1.41 ^a^
6β	1.28 ^a^	7	1.10 ^a^ 1.30 1.30 ^a^	1.11 ^a^ 1.32 1.30 ^a^
7	1.40 ^a^	8	1.33 ^a^	1.32 ^a^
8α	1.09 ^a^1.28	9	1.31 ^a^ 1.36 1.36 ^a^	1.23 ^a^ 1.33 1.32 ^a^
8β	1.28 ^a^	10	1.66 (m)	1.44 (m)
9	1.29 ^a^	11	1.30 ^a^ 1.38 1.38 ^a^	1.23 ^a^ 1.33 1.32 ^a^
10	1.30 ^a^ 1.38 1.38 ^a^	12	1.33 ^a^	1.33 ^a^
11	1.65 (m)	13	1.42 ^a^	1.19 (m)
12	1.36 ^a^	14	-	1.55 (m)
13	1.30 ^a^	15	1.15 (s)	0.89 (d, *J* = 6.4)
14	1.42 ^a^	16	0.94 (d, *J* = 6.6)	0.96 (d, *J* = 6.6)
15	-	17	0.86 (d, *J* = 6.6)	0.88 (d, *J* = 6.4)
16	1.14 (s)	18	3.92 (d, *J* = 5.4)	3.44 (d, *J* = 5.4)
17	0.90 (d, *J* = 6.6)	19	1.15 (s)	0.89 (d, *J* = 6.4)
18	0.85 (d, *J* = 6.6)	-	-	3.79 (dd, *J* = 6.6, 9.4)
19	3.91 (d, *J* = 5.4)	-	-	3.87 (dd, *J* = 6.6, 9.4)
20	1.14 (s)	-	-	-

^a^ Signals overlapped by other resonances.
